# Up-Regulation of LINC00665 Facilitates the Malignant Progression of Prostate Cancer by Epigenetically Silencing KLF2 Through EZH2 and LSD1

**DOI:** 10.3389/fonc.2021.639060

**Published:** 2021-05-20

**Authors:** Peng Xue, Miao Yan, Kunpeng Wang, Jinbao Gu, Bing Zhong, Chuanquan Tu

**Affiliations:** ^1^ Department of Urology, The First People’s Hospital of Lianyungang, Lianyungang Clinical Medical College of Nanjing Medical University, Lianyungang, China; ^2^ Department of Oncology, The First People’s Hospital of Lianyungang, Lianyungang Clinical Medical College of Nanjing Medical University, Lianyungang, China; ^3^ Department of Urology, The Affiliated Huai’an No.1 People’s Hospital of Nanjing Medical University, Huai’an, China

**Keywords:** LINC00665, malignant progression, prostate cancer, KLF2, EZH2, LSD1

## Abstract

This study aimed to explore the function of LINC00665 on the proliferation and metastasis of prostate cancer (PCa), and the potential regulatory mechanisms were also investigated. The expression level of LINC00665 in 50 pairs of PCa tissues and adjacent ones was studied by qRT-PCR, and the associations between LINC00665 and clinicopathological characteristics of PCa patients were analyzed. Control group (sh-NC) and LINC00665 knock-down group (sh-LINC00665) were set in 22RV1 and DU145 cells, respectively. The biological functions of LINC00665 in PCa cell lines were assessed by CCK-8, EdU, Transwell assays, and the nude mouse xenograft model was used to evaluate the tumorigenicity *in vivo*. In addition, qRT-PCR, Western Blot, RIP and ChIP assays were also used to determine the regulation mechanism of LINC00665 in PCa cell lines. In this study, our results showed that LINC00665 expression level in PCa cancer tissues was significantly up-regulated, compared with that in adjacent ones. Besides, similar results were found in PCa cell lines. Knock-down of LINC00665 significantly attenuated the proliferation and migration ability in 22RV1 and DU145 cells, compared to sh-NC. Mechanically, LINC00665 could interact with EZH2 and LSD1, recruiting them to KLF2 promoter region to inhibit its transcription. Moreover, the tumor-suppressive effects mediated by sh-LINC00665 were significantly reversed through the down-regulation of KLF2. Also, the suppression of LINC00665 impaired tumor growth of PCa *in vivo*. In summary, LINC00665 exerted the oncogenic functions in PCa cell lines by epigenetically silencing KLF2 expression by binding to EZH2 and LSD1, illuminating a novel mechanism of LINC00665 in the malignant progression of PCa and furnishing a prospective therapeutic biomarker to combat PCa.

## Introduction

Prostate cancer (PCa) is one of the most common malignancies among men in Western countries and the second most common malignancies in the United States (1, 2). It is reported that 191,930 new cases and 33,330 new deaths of PCa were found among all men in the United States in 2020 (3, 4). The incidence of PCa varies among different ethnic groups, which is relatively low in China compared with Western countries (4). The early symptoms of PCa are insidious, often without any clinical manifestation, and the clinical stage of PCa is often advanced when the clinical symptoms, such as dysuria and urinary retention, and so on, are found (5). However, with the improvement in diagnosis and treatment technology of PCa, the incidence of PCa has gradually decreased in recent years (6). Thus, the early diagnosis and intervention of PCa would help to improve the therapeutic effect on these patients (6, 7). However, despite the continuous in-depth studies, the pathogenesis of PCa is still not fully understood (8, 9). Recently, a large number of studies have focused on exploring the molecular biomarkers that could make up for the defects of the diagnosis of prostate specific antigen (PSA), so as to improve the positive rate of prostate biopsy and avoid unnecessary prostate biopsy for detecting the clinical significant high-grade PCa (10, 11).

With the rapid development of microarray and RNA sequencing technology, non-coding RNAs (ncRNAs) in eukaryote transcription were discovered, which could be divided into long non-coding RNA (LncRNA) and small RNA (siRNA, miRNAs, piRNA) (12, 13). LncRNA is a kind of ncRNA more than 200 nt in length, without the function of encoding proteins (14, 15). Instead, it regulates the expression level of genes at multiple levels in the form of RNA, which was initially considered a “noise” of transcription and was expressed specifically between various cells (15). LncRNAs interact with proteins due to their abundance, location, and diversity, thus creating a complex regulatory network (15, 16). Previous studies have shown that the expression differences of lncRNAs were important mechanisms of the initiation and progression of cancer (16, 17). In the present studies, it has been shown that LINC00665 performed the different biological functions in different types of tumors, and some scholars have reported it could act as an oncogene to promote the proliferation and migration of tumor (18–20). However, the biological functions and molecular mechanisms of LINC00665 in PCa cell lines need to be further investigated. Therefore, it is the focus in identifying the target genes regulated by LINC00665 and its signaling pathways involved.

In this study, we found that LINC00665 expression level was up-regulated in PCa tissues and cell lines. Additionally, the down-regulation of LINC00665 impaired the proliferation and migration of PCa cell lines. Furthermore, LINC00665 epigenetically inhibited the expression level of KLF2 by binding to EZH2 and LSD1, thus promoting the malignant progression of PCa. Together, illuminating the functions and mechanisms of LINC00665 should provide novel insights for the diagnosis and treatment of PCa.

## Materials and Methods

### Patients and PCa Samples

Tumor tissues and adjacent ones of 50 PCa patients undergoing radical prostatectomy were collected in this study. All subjects had not received any preoperative radiotherapy or chemotherapy. All patients were pathologically diagnosed, and the pathological classification and TNM staging criteria of PCa are implemented in accordance with the staging criteria of the International Union Against Cancer (UICC). Each pair of PCa specimens was from the same patient after pathological examination. Then, they were frozen in liquid nitrogen tank for subsequent nucleic acid extraction to conduct molecular biology experiments; and other tissue samples were fixed in 4% formaldehyde for HE staining section to confirm the pathological condition. All PCa patients in this study had fully signed the informed consent form. In addition, this study has been approved by the Ethics Committee of Nanjing Medical University.

### Cell Lines and Reagents

Four human-derived PCa cell lines (PC-3, DU-145, 22RV1, LNCaP) and the human normal prostate stromal immortalized cell line (WPMY-1) provided by ATCC database were cultured in F-12k medium and 1640 medium (Life Technologies, USA) containing 10% fetal bovine serum (10% FBS), 100 U/ml penicillin, and 100 mg/ml streptomycin. These cells were cultured in a humidified air atmosphere at 37°C with 5% CO_2_.

### Transfection

The human PCa cell lines (22RV1 and DU-145) were transfected with LINC00665 knock-down group (sh-LINC00665) or non-specific control sh-RNA (sh-NC) using Lipofectamine 3000 (Invitrogen, Shanghai, China) according to the manufacturer’s protocols. 22RV1 and DU-145 cells were transfected with sh-LINC00665 and sh-NC were used as negative control. For down-regulation of KLF2 (si-KLF2), siRNA targeting the encoding region of KLF2 was purchased from GenePharma (Shanghai, China) and the siRNA transfection reagent (Invitrogen) was used following the manufacturer’s instructions. Non-targeting control siRNA (si-NC) was used as negative control. These cells were collected 48 h later to conduct cell function experiments.

### Proliferation Assay

The proliferation assay was assessed by the cell counting Kit-8 assay (CCK-8, Dojindo, Japan). Pretreated cells were seeded into a 96-well plate with 3 × 10^3^ cells/well and cultured for 1, 2, 3, and 4 days. The absorbance was measured at 450 nm with a microplate reader after incubation at 37°C for 2 h.

### 5-Ethynyl-2′-Deoxyuridine Proliferation Assay

To display the proliferative function of LINC00665 on PCa cell lines, EdU proliferation assay (RiboBio, Nanjing, China) was carried out according to the manufacturer’s instructions. 24 h after the transfection, 22RV1 and DU-145 cells were incubated with 50 μM EdU for 2 h. Then, Apollo staining and DAPI staining were performed according to the instructions to detect EdU positive cells with a fluorescence microscope. The EdU incorporation rate was revealed as the ratio of EdU-positive cells (red cells) to total DAPI-positive cells (blue cells).

### Transwell Migration Assay

Transwell chamber inserts with un-coated Matrigel (migration) were used for the migration assay. The transfected cells (22RV1 and DU-145) were seeded into the upper of the 24-well cell culture insert coating with 200 μl serum-free medium. In addition, 400 μl complete medium supplemented with 10% fetal bovine serum was added into the bottom of the inserts, allowing cells to migrate for 24 h. After incubation, the PCa cells on the upper surface of the membrane were removed, whereas the cells on the lower filter surfaces were fixed and stained with crystal violet. The number of migrated cells was counted under the microscope, according to the manufacturer’s instructions.

### Quantitative Real-Time PCR

In both *in vitro* PCa cell lines and *in vivo* PCa tissue samples, RNA was extracted using an RNeasy Mini Kit (ThermoFisher Scientific, USA) according to the manufacturer’s protocol. 1 ml of TRIzol was used to lyse each sample, and total RNA was extracted. The initially extracted RNA was treated with DNase I to remove genomic DNA and repurify the RNA. RNA reverse transcription was performed according to the Prime Script Reverse Transcription Kit (Takara) instructions. The real-time PCR was performed according to the SYBR^®^ Premix Ex TaqTM (Takara) Kit instructions, and PCR reaction was performed using the StepOne Plus Real-time PCR System (Applied Biosystems, Foster City, CA, USA). Bio-Rad PCR instrument was used to analyze and process the data with software iQ5 2.0. The GAPDH was used as internal parameters, and LINC00665 and KLF2 expressions were calculated by 2-ΔΔCt method. The following primers were used for qRT-PCR reactions:

LINC00665:
forward, 5′-CCAGGTGCAAAGTGGGAAGT-3′,
reverse, 5′-CGGTGGACGGATGAGAAACG-3′;
KLF2:
forward, 5′-GTCTGGAAACCCACCTGGAG-3′,
reverse, 5′-TTGGTGGTCATGGTTACCCG-3′;
GAPDH:
forward, 5′-TGACTTCAACAGCGACACCCA-3′,
reverse, 5′-CACCCTGTTGCTGTAGCCAAA-3′.

### Western Blot

The transfected PCa cells or tissues were lysed using cell lysis buffer, shaken on ice for 30 min, and centrifuged at 14,000 × g for 15 min at 4°C. Total protein concentration was calculated by the BCA Protein Assay Kit (Pierce, Rockford, USA). Anti-KLF2 monoclonal antibodies were purchased from Santa Cruz, USA, while the horseradish peroxidase-labeled goat anti-rabbit secondary antibody was purchased from Genscript. GAPDH was used as internal reference control. Protein samples were separated using SDS-PAGE electrophoresis and then transferred to a PVDF membrane. The membrane was blocked overnight, and then primary antibody and secondary antibody were in turn added for ECL coloration, and the image was semi-quantitatively analyzed by alpha SP image analysis software.

### Subcellular Fractionation Location

PARIS Kit (Life Technologies, Carlsbad, CA, USA) was used to separate the nuclear and cytosolic fractions of PCa cell lines according to the manufacturer’s instructions. The expression levels of LINC00665, GAPDH, and U6 RNA in cytoplasm and nuclear fraction were measured using qRT-PCR assay. GAPDH was used as the cytoplasm control, and U6 was used as the nuclear control.

### RNA Immunoprecipitation

RIP assay was performed to investigate whether LINC00665 could interact with the potential binding protein (EZH2, LSD1, and SUZ12 et al.) in PCa cell lines using Magna RIP™ RNA-Binding Protein Immunoprecipitation Kit (Millipore). Cells were scraped off and lysed in complete RIP lysis buffer. Then, the whole cell extract was incubated with magnetic beads coupled with specific antibodies or control IgG (Cell Signaling Technology). Subsequently, the beads were washed and incubated with Proteinase K to remove the proteins. Finally, the co-precipitated RNAs were purified and analyzed by qRT-PCR.

### Chromatin Immunoprecipitation Assays

ChIP assays were performed using the MagnaChIP Kit (Millipore) according to the manufacturer’s protocol. Cells were cross-linked with 1% formaldehyde, lysed and sonicated to acquire the DNA fragments of 200–500 bp. Then, the chromatin was immunoprecipitated with antibodies against EZH2, LSD1, H3K27me3, H3K4me2, or control IgG (Cell Signaling Technology). Precipitated chromatin DNA was purified and detected by qRT-PCR. The data of ChIP was shown as a percentage relative to input DNA.

### *In Vivo* Xenograft Model

The Animal Ethics and Use Committee of Nanjing Medical University approved the tumor-forming experiment in nude mice. 8-week-old male nude mice were purchased from the animal center of Nanjing Medical University and randomly divided into two groups (five mice in each group). DU-145 cells with sh-LINC00665 and sh-NC were injected subcutaneously into the axilla of mice. Tumor size was monitored every one week; then, after 6 weeks, the mice were sacrificed. The tumor volumes were calculated by the following formula: tumor volume = (width^2^ × length)/2.

### Statistically Analysis

Statistical analysis was performed using SPSS 22.0 software. Univariate analysis was performed using the χ^2^ test and the exact probability Fisher test. Multivariate analysis was performed using COX regression analysis, while the survival of PCa patients was analyzed using the Kaplan–Meier method. Differences between groups were analyzed by the t-test. Pearson correlation test was applied for evaluating the associations between the expression levels of LINC00665 and KLF2 in PCa tissue samples. The data were expressed as mean ± standard deviation, and P <0.05 was considered to be statistically significant.

## Results

### High Expression of LINC00665 in PCa Tissues and Cell Lines

**Figure 1:**
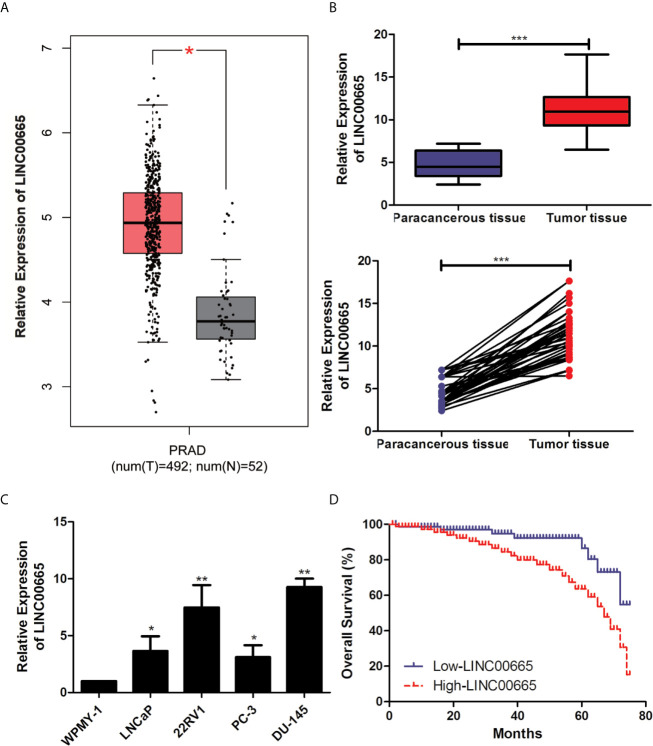
LINC00665 expression level was up-regulated in PCa cell lines and tissues. **(A)** The results of StarBase database showed the high-expression of LINC00665 in PCa tissues. **(B)** qRT-PCR was used to detect the different expression levels of LINC00665 in tumor tissues and corresponding paracancerous ones of PCa patients; **(C)**. qRT-PCR was used to detect the expression level of LINC00665 in PCa cell lines; **(D)**. Kaplan–Meier survival curve of PCa patients based on LINC00665 expression level. Data are mean ± SD, *p < 0.05, **p < 0.01, ***p < 0.001.

The data from PCa patients of StarBase database were compiled for investigating the expression level of LINC00665 associated with the malignant progression of PCa. Firstly, we focused the insight into the expression level of LINC00665 from the StarBase database. As shown in **Figure 1A**, the expression level of LINC00665 significantly up-regulated in PCa tissues, compared with that in adjacent ones (P < 0.05). Then, to explore the function of LINC00665 in PCa patients, the result of qRT-PCR analysis of PCa patients was consistent with the analysis result from the StarBase database (**Figure 1B**, P < 0.05). Meanwhile, qRT-PCR also revealed the up-regulated expression level of LINC00665 in PCa cell lines, particularly in 22RV1 and DU-145 cells, which were used for subsequent cell function experiments (**Figure 1C**).

### High Expression of LINC00665 Was Correlated With the Higher T Stage and Lymph Node Metastasis of PCa Patients

50 pairs of tumor tissues and corresponding paracancerous ones were collected from these PCa patients and were divided into LINC00665 high-expression group and LINC00665 low-expression group. The associations of LINC00665 expression level with Age, Serum PSA, Gleason score, Tumor size, T stage and Lymph node metastasis of PCa patients were further explored in this study. **Table 1** indicated that the patients in the LINC00665 high-expression showed higher T stage and lymph node metastasis of PCa patients in comparison to the PCa patients in the LINC00665 low-expression. Then, Kaplan–Meier survival analysis was performed to access the associations between LINC00665 expression level and the prognosis of PCa patients, and the results demonstrated that high LINC00665 expression level was dramatically correlated with the poor survival of PCa patients. The 5 years overall survival rate of the PCa patients with high LINC00665 expression (n = 24) was lower than that of the patients with low LINC00665 expression (n = 26) (P < 0.05, **Figure 1D**). Taken together, these results suggested that LINC00665 might be involved in the malignant progression of PCa. 

**Table 1:** Association of LINC00665 expression with clinicopathologic characteristics of prostate cancer.

Variables	Patient number(N)	LINC00665 expression		*P*-value
		Low (n = 26)	High (n = 24)	
Age (years)				0.160
≤60	24	10	14	
>60	26	16	10
Serum PSA (ng/ml)				0.555
≤10	27	13	14	
>10	23	13	10
Gleason score				0.144
≤6	22	14	8	
>6	28	12	16
Tumor size (cm)				0.333
≤2	32	15	17	
>2	18	11	7
T stage				0.011*
T1-2	28	19	9	
T3-4	22	7	15
Lymph node metastasis				0.011*
No	30	20	10	
Yes	20	6	14

Low/high by the sample median. Pearson χ2 test.

*P < 0.05 was considered statistically significant. The bold values was statistical difference between the two groups.

### Knock-Down of LINC00665 Inhibited the Proliferation and Migration in PCa Cell Lines

**Figure 2:**
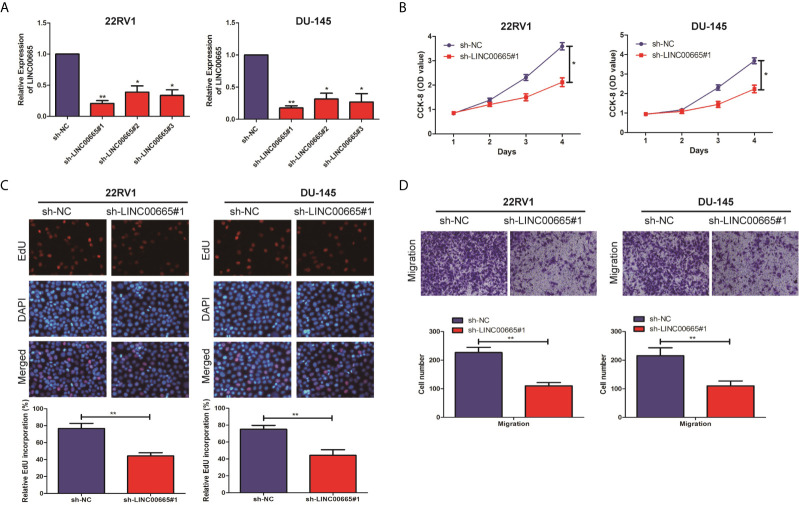
LINC00665 promoted the proliferation and migration of PCa cell lines. **(A)** qRT-PCR verified the transfection efficiency of LINC00665 knockdown vector in PCa cell lines (22RV1 and DU145); **(B)** CCK-8 assay detected the proliferation of PCa cells in the transfection of LINC00665 knockdown vector; **(C)** EdU assay was used to detect the proliferation ability after the transfection of LINC00665 knock-down vectors in 22RV1 and DU145 cells (Magnification: 40×); **(D)**. Transwell assay was used to detect the migration ability after the transfection of LINC00665 knock-down vectors in 22RV1 and DU145 cells (Magnification: 40×). Data are average ± SD, *p < 0.05, **p < 0.01.

To explore, the biological function of LINC00665 in PCa cell lines was analyzed by Cell proliferation assay, EdU assay, and Transwell assay. The knockout vectors of LINC00665 were successfully constructed in 22RV1 and DU-145 cell lines, respectively (**Figure 2A**). It was found by the CCK-8 assay that the cell proliferation ability of sh-LINC00665 was significantly decreased in PCa cell lines compared with sh-NC (**Figure 2B**). EdU assay suggested that knock-down of LINC00665 has lower proliferation capabilities than sh-NC in 22RV1 and DU-145 cells (**Figure 2C**). Transwell assay revealed that the metastasis ability of PCa cell lines was significantly decreased in sh-LINC00665 compared with sh-NC (**Figure 2D**). These results suggested that LINC00665 could increase proliferation and metastasis in PCa.

### LINC00665 Exerted Oncogenic Activity by Silencing KLF2 in PCa Cells

**Figure 3:**
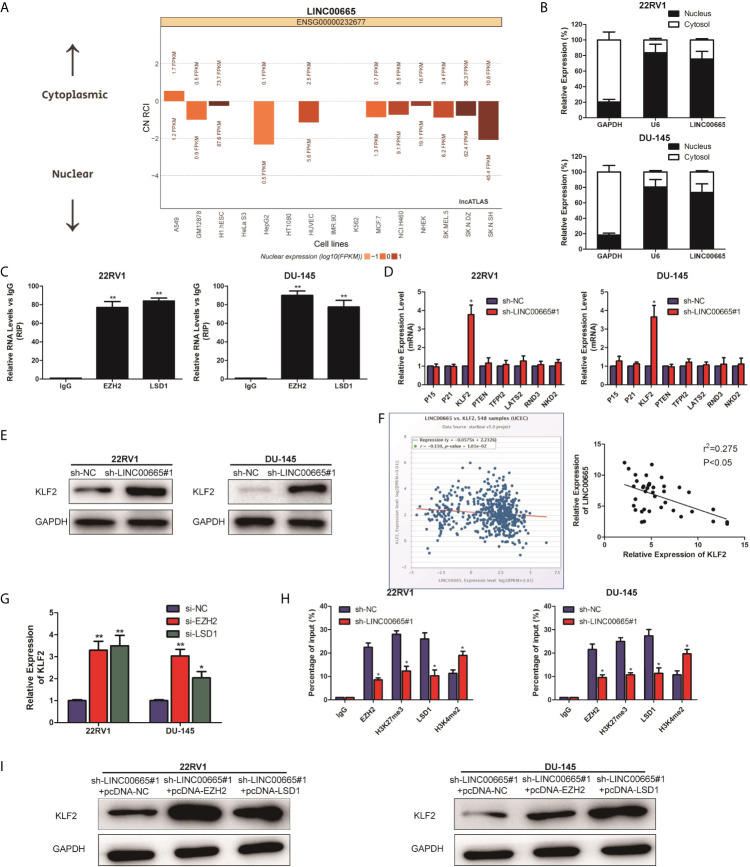
LINC00665 recruited EZH2 and LSD1 to suppress KLF2 expression. **(A)** The subcellular localization of LINC00665 predicted on lncATLAS website; **(B)** qRT-PCR analysis detected LINC00665 expression level in the nuclear and cytoplasmic fraction of 22RV1 and DU145 cells. GAPDH and U6 were used as the cytoplasm marker and nucleus marker, respectively. **(C)** RIP experiments were conducted in 22RV1 and DU145 cells using antibodies against EZH2 and LSD1, followed by qRT-PCR assay of LINC00665 expression level in immunoprecipitates. The fold enrichment was relative to IgG immunoprecipitate. **(D)** qRT-PCR analysis was performed to evaluate the levels of P15, P21, KLF2, PTEN, KLF2, LATS2, RND3, and NKD2 in sh-NC or sh-LINC00665-transfected 22RV1 and DU145 cells. **(E)** Western Blot analysis detected KLF2 expression level in 22RV1 and DU145 cells following knock-down of LINC00665. **(F)** A significant negative correlation between LINC00665 and KLF2 expression was found in PCa tissues. **(G)** KLF2 expression levels at mRNA levels were detected in 22RV1 and DU145 cells after transfection with si-EZH2 or si-LSD1. **(H)** ChIP assay showed EZH2/LSD1 occupancy and H3K27me3/H3K4me2 binding in the KLF2 promoter in 22RV1 and DU145 cells after transfection with sh-NC or sh-LINC00665. Enrichment was quantified relative to input control. IgG was used as a negative control. **(I)** The expression level of KLF2 in the co-transfected PCa cell lines of sh-LINC00665 and pcDNA-EZH2/LSD1 was detected by Western Blot. Data are average ± SD, *p < 0.05, **p < 0.01.

It is well known that lncRNAs are able to regulate cell phenotypes through interacting with specific RNA-binding proteins. As predicated by the lncATLAS website (http://lncatlas.crg.eu/), LINC00665 was mainly predicted to be localized in the nuclei (**Figure 3A**). To examine the potential biological mechanisms of LINC00665 involved in PCa cell lines, subcellular fractionation assays were performed to determine the distribution of LINC00665 in nuclear and cytoplasmic fractions in PCa cells. The results revealed that LINC00665 was mainly located in the nucleus of 22RV1 and DU-145 cells (**Figure 3B**), indicating that LINC00665 might exert regulatory effects at transcriptional levels. Then, RIP assays were used to analyze the possible RNA-binding proteins of LINC00665 in PCa cell lines. As presented in **Figure 3C**, LINC00665 could directly bind with EZH2 and LSD1 in 22RV1 and DU-145 cells. Next, we selected several EZH2 or LSD1 potential targets (P15, P21, KLF2, PTEN, KLF2, LATS2, RND3, and NKD2) with tumor-suppressive role, and hypothesized that some of which might be associated with LINC00665-mediated carcinogenicity. The results of qRT-PCR disclosed that KLF2 expression was significantly up-regulated in both 22RV1 and DU-145 cells after the knock-down of LINC00665 (**Figure 3D**). In addition, we demonstrated by Western Blot assay that the expression level of KLF2 was significantly up-regulated after the knock-down of LINC00665 (**Figure 3E**). Furthermore, we confirmed a negative correlation of LINC00665 and KLF2 in PCa tissues by applying qRT-PCR in the StarBase database (**Figure 3F**). We also validated the expression level of LINC00665 and KLF2 in PCa tissues and obtained a negative correlation between the expression level of LINC00665 and KLF2 (P < 0.05, **Figure 3F**). Meanwhile, the knock-down of EZH2 or LSD1 resulted in the up-regulation of KLF2 expression at mRNA levels (**Figure 3G**). These results implied that KLF2 might be a downstream mediator of LINC00665. To further address whether LINC00665 inhibited KLF2 expression through interacting with EZH2 and LSD1, ChIP analysis was conducted in 22RV1 and DU-145 cells, which illuminated that LINC00665 could recruit EZH2 and LSD1 to the KLF2 promoter region, resulting in trimethylation of H3K27 or demethylation of H3K4 at this region (**Figure 3H**). To determine EZH2 and LSD1 as the downstream of LINC00665, PCa cells with sh-LINC00665 were transfected with pcDNA-EZH2 and pcDNA-LSD1. After transfection, Western Blot analysis demonstrated that KLF2 expression was significantly up-regulated in 22RV1 and DU-145 cells co-transfected with sh-LINC00665 and pcDNA-EZH2/pcDNA-LSD1 compared with sh-LINC00665 + pcDNA-NC/pcDNA-NC (**Figure 3I**).

### Down-Regulation of KLF2 Reversed the Tumor-Suppressive Effects Mediated by LINC00665 Knockdown in PCa Cell Lines

**Figure 4:**
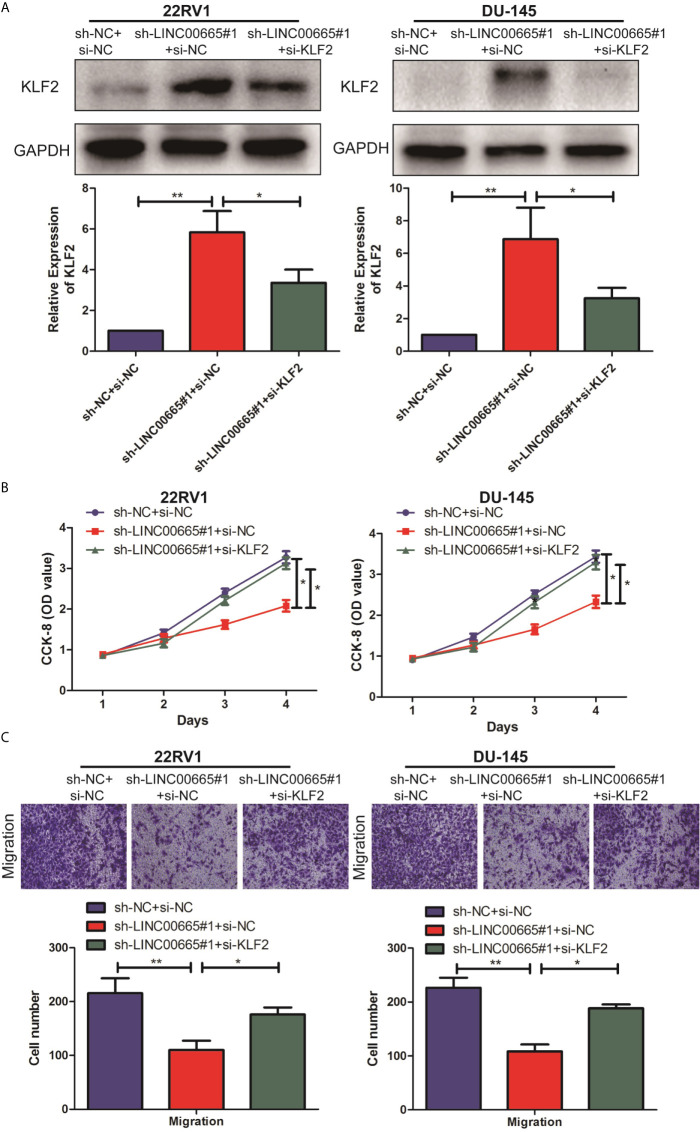
LINC00665 regulated the expression level of KLF2 in PCa cell lines. **(A)** The expression level of KLF2 in the co-transfected PCa cell lines of LINC00665 and KLF2 was detected by Western Blot and qRT-PCR; **(B)** CCK-8 assay detected the proliferation ability in the co-transfected PCa cell lines of sh-LINC00665 and si-KLF2; **(C)** Transwell assay was used to detect the migration of PCa cell lines after co-transfection of sh-LINC00665 and si-KLF2 (Magnification: 40×). Data are average ± SD, *p < 0.05, **p < 0.01.

We further explored the specific regulatory mechanisms in which LINC00665 exactly regulated KLF2 to promote malignant progression of PCa. Firstly, PCa cell lines with sh-LINC00665 were transfected with si-KLF2 or si-NC to downregulate endogenous KLF2. After siRNA transfection, qRT-PCR analysis demonstrated that KLF2 expression was significantly down-regulated in 22RV1, and DU-145 cells transfected with si-KLF2 (**Figure 4A**). Subsequently, the knock-down of KLF2 was demonstrated to be able to counteract the proliferation and metastasis effects of sh-LINC00665 in PCa cells by CCK-8 and Transwell assays (**Figures 4B, C** **)**. Therefore, these results revealed that LINC00665 could promote the malignant progression of PCa through modulating KLF2.

### Knockdown of LINC00665 Inhibited PCa Tumorigenesis *In Vivo*

**Figure 5:**
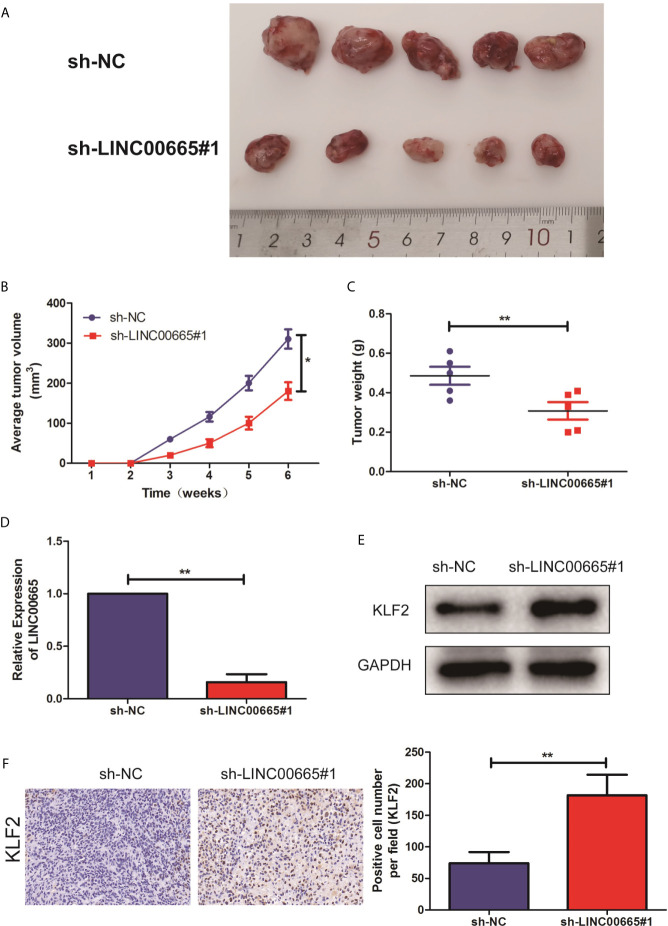
Knock-out of LINC00665 inhibited tumorigenic ability in nude mice. **(A)** Representative pictures of tumor in sh-NC or sh-LINC00665 stably transfected DU-145 cells. **(B)** Tumor volume growth curves were calculated for different nude mice after injection of sh-NC and sh-LINC00665, respectively; **(C)** Tumor weight growth curves were calculated after injection of sh-NC and sh-LINC00665, respectively; **(D)** qRT-PCR was used to detect the expression level of LINC00665 in the tumor-forming tissues of nude mice; **(E)** Western Blot was used to detect the expression level of KLF2 in the tumor-forming tissues of nude mice; **(F)** Immunohistochemistry was used to detect the expression level of KLF2 in the tumor-forming tissues of nude mice with DU145 cells (Magnification: 40×). Data are mean ± SD, *p < 0.05, **p < 0.01.

To further address the biological significance of LINC00665 in the tumor growth *in vivo*, sh-NC or sh-LINC00665 stably transfected DU-145 cells were subcutaneously injected into the left flank of nude mice. The results showed that silencing of LINC00665 significantly decreased the tumor growth (**Figures 5A, B** **)**. Moreover, the tumor weights of nude mice were decreased in sh-LINC00665-transfected DU-145 cells (**Figure 5C**). Additionally, the knock-down of LINC00665 triggered a reduction of LINC00665 expression (**Figure 5D**) while an increase of KLF2 protein level (**Figure 5E**) in excised tumor masses. In addition, the immunohistochemistry showed that the KLF2 expression level of sh-LINC00665-transduced DU-145 xenografts significantly increased than sh-NC-transduced xenografts (**Figure 5F**).

## Discussion

PCa, as one of the most common malignant tumors, has the second highest incidence and the sixth highest mortality worldwide (1, 2) Although the incidence of PCa in China is far less likely to that in Western countries, its growth in the developing country is much faster (3, 4). As China gradually enters the aging society, PCa will become the main tumor “killer” that endangers the health of elderly men; therefore, the clinical diagnosis and treatment of PCa and its basic study have entered an urgent stage (5). Non-coding RNAs (ncRNAs) were originally thought to be transcription noise and also exhibited its important functions (12, 13). LncRNA, a type of ncRNA with a transcript longer than 200 nt, does not encode proteins or only encodes short peptides (14, 15) A large number of studies have revealed that lncRNA plays a non-negligible role in the development of PCa (21, 22). Prostate cancer antigen 3 (PCA3), as one of the LncRNAs, has been proven to be a diagnostic marker to improve the diagnostic efficacy of PSA in PCa and has been approved by the FDA to guide repeated prostate biopsy (23, 24). The lncRNA sequence contains nucleic acid binding sites and can be folded to form higher-level structures with corresponding protein-binding sites, which interacts with DNA, RNA, and proteins to affect the regulation of body functions (15, 16). Various characteristics of lncRNA, such as the extensive tissue expression profile and high specificity of tissue and cell, make it a good prospect for the molecular diagnosis and targeted therapy of tumor (16, 17). Based on the next-generation sequencing of PCa tissues to an in-depth exploration of PCa tumor-related lncRNAs in the Chinese population, these studies focused on the molecular characteristics and functional mechanisms to better understand the biological behaviors of PCa cells, find new targets for the treatment of PCa, and provide evidence for the clinical diagnosis and new drug development of PCa (25, 26).

LINC00665 has been discovered for an extended period of time and is engaged in many physiological and pathological processes including the progression of tumors (18–20). In this study, to explore the function of LINC00665 in the malignant progression of PCa, we performed qRT-PCR assay and detected the up-regulated LINC00665 expression level in PCa tissues. Meanwhile, we found that LINC00665 could be used as an indicator of the proliferation ability and thus serve as an oncogene in PCa. Furthermore, Transwell assays were carried out to reveal that LINC00665 could enhance the migratory capacity of PCa cell lines. However, the specific molecular mechanism of LINC00665 still remains unclear.

To our knowledge, one of the mechanisms of lncRNAs is to recruit proteins or RNAs to target genes, thus exerting its biological significance indirectly (16, 27). To clarify the possible mechanism of LINC00665 involved in the pathogenesis of PCa, subcellular fractionation assays were performed. As a result, LINC00665 was found to be mostly located in the nucleus fractions of 22RV1 and DU-145 cells, reflecting its regulation at the transcriptional level. Subsequently, RIP assay verified that LINC00665 could directly bind to EZH2 and LSD1. Aberrant high expression levels of EZH2 and LSD1 have been reported to be associated with multiple cell processes in many malignancies. By qRT-PCR and Western Blot analysis, we found the increase of KLF2 mRNA and protein levels in 22RV1 and DU-145 cell lines following knock-down of LINC00665. Similarly, the elevated KLF2 mRNAs were observed in EZH2 and LSD1 knock-down 22RV1 and DU-145 cells. Besides, EZH2 or LSD1 overexpression further increased KLF2 protein expression level in PCa cells with sh-LINC00665. That is to say, KLF2 is a novel target of LINC00665 in PCa cells. However, its regulatory mechanism is still unclear. EZH2 is a negative regulator of transcription *via* histone 3 lysine 27 trimethylation (H3K27me3), whereas LSD1 is a negative regulator of transcription *via* histone 3 lysine 4 demethylation (H3K4me2). Furthermore, ChIP assays elucidated that LINC00665 could recruit EZH2 and LSD1 to KLF2 promoter region and suppress their transcription through mediating H3K27me3 and H3K4me2 modifications. To conclude, LINC00665 epigenetically silenced KLF2 expression in PCa cell lines *via* binding to EZH2 and LSD1.

Evidence from bioinformatics research indicates that specific LncRNAs have the potential to recruit proteins or RNAs to target genes and are likely to play a role in the splicing process of target gene miRNA (16, 28). Hence, to determine the biological functions of LINC00665, it is necessary to further find its target genes. The up-regulated expression level of LINC00665 in PCa tissues and cell lines was detected, which was negatively correlated with that of KLF2. Furthermore, to investigate the impact of the interaction between LINC00665 and KLF2 in the development of PCa, reverse experiments were performed *in vitro* and *in vivo*. It was found that knock-down of KLF2 could counteract the tumor-suppressive effect of the knock-down of LINC00665 on the proliferation and migration ability of PCa cell lines.

## Conclusion

In summary, we first revealed that LINC00665 functioned as a proliferative and migrative lncRNA in PCa both *in vitro* and *in vivo*. Our research defined a molecular mechanism for the induction function of LINC00665 by epigenetically silencing KLF2 through EZH2 and LSD1. The identification and validation of our study provided a novel insight into research and clinical management of PCa. Therefore, this newly identified LINC00665 might serve as a prognostic biomarker and a potential therapeutic target for PCa.

## Data Availability Statement

The original contributions presented in the study are included in the article/supplementary material. Further inquiries can be directed to the corresponding authors. 

## Ethics Statement

The studies involving human participants were reviewed and approved by Nanjing Medical University. The patients/participants provided their written informed consent to participate in this study. The animal study was reviewed and approved by Nanjing Medical University. Written informed consent was obtained from the individual(s) for the publication of any potentially identifiable images or data included in this article.

## Author Contributions

Protocol/project development: BZ and CT. Molecular biology experiment: PX and KW. Data collection or management: KW and JG. Data analysis: MY and KW. Manuscript writing/editing: PX and MY. All authors contributed to the article and approved the submitted version.

## Conflict of Interest

The authors declare that the research was conducted in the absence of any commercial or financial relationships that could be construed as a potential conflict of interest.
